# A novel transferrin receptor-targeted hybrid peptide disintegrates cancer cell membrane to induce rapid killing of cancer cells

**DOI:** 10.1186/1471-2407-11-359

**Published:** 2011-08-18

**Authors:** Megumi Kawamoto, Tomohisa Horibe, Masayuki Kohno, Koji Kawakami

**Affiliations:** 1 Department of Pharmacoepidemiology, Graduate School of Medicine and Public Health, Kyoto University, Kyoto, Japan; 2 Department of R & D Promotion, Upstream Infinity, Inc., 1-7-8 Kaigan, Minato-ku, Tokyo 105-0022, Japan

## Abstract

**Background:**

Transferrin receptor (TfR) is a cell membrane-associated glycoprotein involved in the cellular uptake of iron and the regulation of cell growth. Recent studies have shown the elevated expression levels of TfR on cancer cells compared with normal cells. The elevated expression levels of this receptor in malignancies, which is the accessible extracellular protein, can be a fascinating target for the treatment of cancer. We have recently designed novel type of immunotoxin, termed "hybrid peptide", which is chemically synthesized and is composed of target-binding peptide and lytic peptide containing cationic-rich amino acids components that disintegrates the cell membrane for the cancer cell killing. The lytic peptide is newly designed to induce rapid killing of cancer cells due to conformational change. In this study, we designed TfR binding peptide connected with this novel lytic peptide and assessed the cytotoxic activity *in vitro *and *in vivo*.

**Methods:**

*In vitro*: We assessed the cytotoxicity of TfR-lytic hybrid peptide for 12 cancer and 2 normal cell lines. The specificity for TfR is demonstrated by competitive assay using TfR antibody and siRNA. In addition, we performed analysis of confocal fluorescence microscopy and apoptosis assay by Annexin-V binding, caspase activity, and JC-1 staining to assess the change in mitochondria membrane potential. *In vivo*: TfR-lytic was administered intravenously in an athymic mice model with MDA-MB-231 cells. After three weeks tumor sections were histologically analyzed.

**Results:**

The TfR-lytic hybrid peptide showed cytotoxic activity in 12 cancer cell lines, with IC_50 _values as low as 4.0-9.3 μM. Normal cells were less sensitive to this molecule, with IC_50 _values > 50 μM. Competition assay using TfR antibody and knockdown of this receptor by siRNA confirmed the specificity of the TfR-lytic hybrid peptide. In addition, it was revealed that this molecule can disintegrate the cell membrane of T47D cancer cells just in 10 min, to effectively kill these cells and induce approximately 80% apoptotic cell death but not in normal cells. The intravenous administration of TfR-lytic peptide in the athymic mice model significantly inhibited tumor progression.

**Conclusions:**

TfR-lytic peptide might provide a potent and selective anticancer therapy for patients.

## Background

The transferrin receptor (TfR) is a cell-membrane-associated glycoprotein involved in the cellular uptake of iron and the regulation of cell growth [1]. Iron is a required cofactor of heme and nonheme proteins involved in a variety of cellular processes including metabolism and DNA synthesis [2,3]. Therefore, various studies have shown elevated levels of TfR expression on cancer cells when compared with their normal counterparts [4-13]. Bladder-transitional cell carcinomas, breast cancer, glioma, lung adenocarcinoma, chronic lymphocytic leukemia, and non-Hodgkin's lymphoma also showed increased TfR expression that correlated with tumor grade and stage or prognosis [8,9,11-14]. These data suggest that TfR expression may be increased on circulating tumor cells, tumor precursor cells, or cells that have been activated during tumorigenesis [15]. The elevated levels of TfR in malignancies, its relevance in cancer, and the extracellular accessibility of this molecule make it an ideal candidate for the targeting of cancer cells.

Immunotoxins are chimeric proteins with a cell-selective ligand chemically linked or genetically fused to a toxin moiety. They can target cancer cells overexpressing tumor-associated antigens, membrane receptors, or carbohydrate antigens [16,17]. Generally, ligands for these receptors, monoclonal antibodies, or single-chain variable fragments directed against these antigens are fused with bacterial or plant toxins to generate immunotoxins. Several such fusion proteins including *Pseudomonas *exotoxin-based interleukin-4-*Pseudomonas *exotoxin (IL4(38-37)-PE38KDEL) and interleukin-13-*Pseudomonas *exotoxin (IL13-PE38QQR) fusion proteins have been tested in clinical trials [18,19]. Interleukin-2-diphtheria toxin fusion protein (IL2-DT; Ontak™) is an FDA-approved fusion protein [20,21].

However, bacterial- or plant-toxin-based chimeric proteins pose several obstacles that limit their clinical applications [22], since the toxin part of these fusion proteins elicits a high degree of humoral response in the human body. Besides, in developed countries where people are immunized against diphtheria, human serum will contain circulating antibodies against diphtheria toxin, which will result in neutralization of diphtheria-toxin-based immunotoxins [23,24]. At sufficiently high concentrations these fusion proteins also lead to vascular leak syndrome and show some degree of non-specific toxicity. In addition, the molecular size of these immunotoxins is generally greater than chemical compounds or fragment antibody drugs, which might prevent drugs from efficiently penetrating into larger tumor masses in the human body.

As a new generation of immunotoxins, we have generated a chemically synthesized hybrid peptide, which is composed of target-binding and cell-killing sequence components. Papo and Shai [25] reported a new artificial cell-membrane-lytic peptide which kills tumor cells better than normal cells. However, when this peptide was fused to a molecular-targeted sequence the selectivity for cancer cells decreased considerably [25]. These results prompted us to design a new lytic-type sequence which is suitable for connecting to a molecular-targeted sequence [26].

Utilizing a new lytic-targeted sequence, in this study we designed a novel peptide targeting TfR, termed TfR-lytic hybrid peptide, which is composed of a TfR-binding moiety and a cellular-membrane-lytic moiety using the previous identification of peptide sequences binding to TfR [27]. We demonstrated the selective cytotoxic activity and characterized the cancer-cell-killing mechanisms of this molecule, and, moreover, assessed the antitumor activity of TfR-lytic peptide in xenograft model *in vivo*.

## Methods

### Cell lines

Human breast cancer (BT474, T47D, SK-BR-3, MDA-MB-231, BT20, and ZR75-1), glioblastoma (SN-19, and U251), prostate cancer (LNCap), and pancreatic cancer (COLO587) cell lines, were purchased from the American Type Culture Collection (Manassas, VA, USA). Bile-duct cancer cells (HuCCT-1) were purchased from Health Science Research Resources Bank (Osaka, Japan). Glioblastoma (SF295) was obtained from the National Institutes of Health (Bethesda, MD, USA). Primary hepatocyte ACBRI 3716 (HC) and pancreatic epithelial ACBRI 515 (PE) cells were purchased from the European Collection of Cell Culture (Salisbury, UK). Cells were cultured in RPMI-1640 (BT474, T47D, MDA-MB-231, BT20, ZR75-1, SF295, SN-19, U251, LNCap, COLO587, and HuCCT-1), CS-C (HC and PE), or McCoy's 5A (SK-BR-3) - with 10% FBS (BioWest, Miami, FL, USA), 100 μg/ml penicillin, and 100 μg/ml streptomycin (Nacalai Tesque, Kyoto, Japan) added to all media - under 5% CO_2_.

### Peptides preparation and synthesis

The following peptides were purchased from Invitrogen (Carlsbad, CA, USA) or SIGMA (St Louis, MO, USA). Note that in both cases bold and underlined letters indicate D-amino acids.

1. Lytic peptide: KL**L**LK**L**L**KK**LLK**L**LKKK

2. Transferrin receptor (TfR)-lytic hybrid peptide:

THRPPMWSPVWPGGGKL**L**LK**L**L**KK**LLK**L**LKKK

All peptides were synthesized by use of solid-phase chemistry, purified to homogeneity (i.e. > 80%) by reversed-phase high-pressure liquid chromatography, and assessed by mass spectrometry. Peptides were dissolved in water.

### Cell viability assay

A total of 3 × 10^3 ^cells per well were seeded into 96-well plates and incubated for 24 h in medium containing 10% FBS. The cells were then incubated with increasing concentrations of lytic peptide or the TfR-lytic peptide in 100 μl of medium for 72 h at 37°C. Cell viability was measured using WST-8 solution (Cell Count Reagent SF, Nacalai Tesque). For competition assays, the cells were incubated with anti-TfR monoclonal antibody (eBiosience) or mouse IgG isotype control for 3 h then incubated with TfR-lytic hybrid peptide.

### Immunofluorescence staining

TfR expression was determined using flow cytometry by incubating 1 × 10^6 ^cells with a PE-conjugated human monoclonal antibody to TfR (BD Biosciences San Jose, CA, USA). All staining was performed at room temperature for 1.5 h. The cell fluorescence was measured by flow cytometry (FACS Calibur, BD, Biosciences San Jose, CA, USA). The value of mean fluorescence intensity (MFI) was determined using Win MDI version 2.9 software.

### Confocal fluorescence microscopy

T47D cells were grown to 20% confluence on a glass-bottomed dish for 24 h with a soluble fluorescent molecule, calcein, which was added to the medium at a final concentration of 5 μM. Confocal images were taken after 5, 10, and 15 min after addition of TfR-lytic peptide using an Olympus FV1000 confocal laser scanning microscope (Olympus, Tokyo, Japan).

### siRNA transfection

The following stealth RNA duplexes were synthesized by Invitrogen:

TfR sense, 5'-AACAGAAAGAAACUGCUGGGAUUCC-3',

TfR antisense, 5'-GGAAUCCCAGCAGUUUCUUUCUGUU-3';

scramble sense, 5'-GCAUCGUACAGACAAUCUUCAGUUU-3', and

scramble antisense, 5'-AAACTGAAGAUUGUCUGUACGAUGC-3'.

T47D and MDA-MB-231 cells were grown to 40% confluence on a six-well plate, and then transfection of these cells with siRNAs (100 pmol/ml) was performed using Lipofectamine RNAi MAX (Invitrogen), according to the manufacturer's protocol.

### Flow cytometry

T47D, and PE cells were treated for 3 h at 37°C with or without TfR-lytic hybrid peptide or lytic peptide at 10 μM. For annexin V-propidium iodide (PI) and caspase 3&7-PI staining, cells were centrifugated and washed in PBS. Then peptide-treated cultures were simultaneously analyzed for annexin V-PI staining using the Annexin V-Fluorescein Staining Kit (Wako, Osaka, Japan) and caspase 3&7-PI staining using a carboxyfluorescein FLICA caspase 3&7 assay (Immunochemistry Technologies, Bloomington, MN, USA), by flow cytometry.

To analyse mitochondrial membrane potential, T47D cells were labeled for 10 min with the mitochondrial-membrane-potential-sensitive fluorescent dye JC-1 (BioVision Inc., Mountain View, CA, USA). After washing in PBS samples were analyzed by flow cytometry to monitor changes in the red/green fluorescence ratio to obtain the index of mitochondrial membrane depolarization.

### *In vivo *efficacy in xenograft models

Animal experiments were carried out in accordance with the guidelines of Kyoto University School of Medicine. Cells of the breast cancer cell line MDA-MB-231 (5 × 10^6^cells), resuspended in 150 μl of PBS, were transplanted subcutaneously into the flank region of 6-9-week-old athymic female nude mice weighing 17-21 g. When tumours reached 20-60 mm^3 ^in volume, animals were randomised into two groups, and saline (control) or TfR-lytic peptide (3 mg/kg) was injected intravenously (50 μl/injection) three times a week for a total of nine doses. Tumours were measured with a caliper and the tumour volume (in mm^3^) was calculated using the following formula: length × width^2 ^× 0.5. All values are expressed as the mean ± SD.

## Results

The cytotoxic activity of TfR-lytic hybrid peptide is dependent on cell-surface TfR expression levels

**Figure 1:**
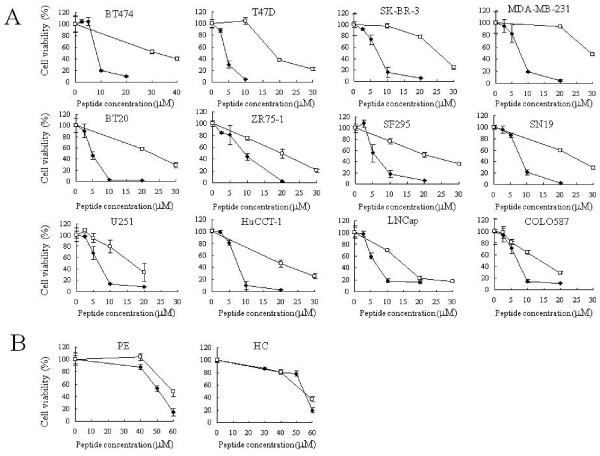
**The cytotoxic activity of TfR-lytic hybrid peptide**: (A) Twelve cancer cell lines were cultured with various concentrations of TfR-lytic hybrid peptide or lytic peptide (0-30 μM) for 72 h, and cytotoxic activity was assessed using WST-8 reagent. (B) Two normal cell lines were cultured with various concentrations of peptides (0-60 μM) for 72 h and cytotoxic activity was assessed. Absorbance values obtained with untreated cells were set at 100%. Black and white squares indicate TfR-lytic hybrid peptide and lytic peptide, respectively. Data are represented by means ± SD (bars) from triplicate determinations.

We first examined the correlation between cytotoxic activity and the expression levels of TfR on the cell surface using 12 cancer and 2 normal cell lines. As shown in Figure 1A, treatment with the lytic peptide alone induced dose-dependent cytotoxic cell killing in all cancer cell lines. However, molecular targeted TfR-lytic hybrid peptide was superior in inducing cytotoxic activity in all cell lines. As shown in Table 1, the range of IC_50 _values (the peptide concentration inducing 50% inhibition of control cell growth) were 4.0-9.3 μM for TfR-lytic hybrid peptide, whereas lytic peptide alone induced modest cytotoxic activity with the IC_50 _value ranging from 13.5 to 34.5 μM. A TfR-lytic hybrid peptide concentration of merely 15-20 μM was sufficient to induce more than 80% cell death in all cancer cell lines examined (Figure 1A). These data suggest that cancer cells are more susceptible (2.0-4.2-fold) to the TfR-lytic hybrid peptide than to the lytic peptide alone.

**Table 1:** Cytotoxicity of peptides to various cell lines and TfR expression

Cell line		IC_50_ Lytic peptide alone	(μM)** TfR-lytic peptide	IC_50_Ratio Lyttic/TfR-lytic	MFI* mean	Statistical Difference vs HC
					
		mean	mean			
*Cancer cells*	*Organ*					
**BT474**	**Breast**	34.5 ± 3.8	8.8 ± 3.3	3.9	99.9 ± 24.9	< 0.05
**T47D**	**Breast**	14.1 ± 3.6	4.0 ± 0.2	3.6	101.4 ± 32.4	< 0.05
**SK-BR-3**	**Breast**	25.7 ± 0.6	6.5 ± 0.9	4.0	79.7 ± 21.2	< 0.05
**MDA-MB-231**	**Breast**	27.0 ± 1.5	7.8 ± 0.8	3.4	98.1 ± 23.9	< 0.05
**BT20**	**Breast**	20.0 ± 2.9	4.8 ± 0.4	4.2	91.5 ± 14.6	< 0.05
**ZR75-1**	**Breast**	19.4 ± 1.7	9.3 ± 2.6	2.1	46.4 ± 9.0	0.08
**SF295**	**Brain**	19.1 ± 2.9	6.3 ± 1.8	3.0	56.9 ± 17.6	0.08
**SN19**	**Brain**	21.9 ± 4.0	7.8 ± 0.5	2.8	75.3 ± 10.0	< 0.05
**U251**	**Brain**	17.0 ± 1.4	7.0 ± 2.0	2.4	56.3 ± 22.8	0.13
**HuCCT-1**	**Bile duct**	17.1 ± 2.8	8.3 ± 2.5	2.0	46.2 ± 4.6	< 0.05
**LNCap**	**Prostate**	15.8 ± 1.5	6.2 ± 0.0	2.5	57.8 ± 5.9	< 0.05
**COLO587**	**Pancreas**	13.5 ± 1.2	6.4 ± 1.3	2.1	46.4 ± 15.8	0.18

*Normal cells*						
**Hepatocyte (HC)**	**Liver**	59.2 ± 5.0	54.7 ± 3.8	1.1	35.5 ± 4.2	
**Pancreatic epithelical (PE) cell**	**Pancreas**	59.6 ± 7.2	51.2 ± 3.6	1.2	2.8 ± 0.1	

*MFI, mean fluorescence intensity; this is the extent of binding of the PE-conjugated anti-TfR monoclonal antibody to cells. The value of MFI is represented by means ± SD from independent 3 time assays.

**The value of IC_50 _is represented by means ± SD from triplicate determinations, and the assay was repeated three times.

We then assessed the cytotoxic activity of these peptides in two normal cell lines. As shown in Figure 1B, two normal cell lines, HC and PE, hardly showed any cell death at a peptide concentration of 40 μM, indicating that these normal cells were less sensitive to the peptides than cancer cell lines.

**Figure 2:**
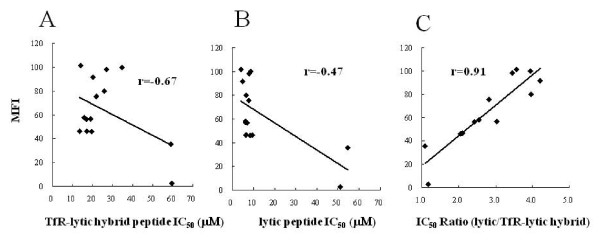
**Enhancement of lytic-peptide cytotoxic activity by addition of the TfR-targeting moiety**: IC_50 _values of TfR-lytic hybrid peptide (A) and lytic peptide alone (B) in normal and cancer cells, and the IC_50 _ratio of lytic/TfR-lytic hybrid peptide (C), are shown plotted against the mean fluorescence intensity (MFI) of TfR expression.

The correlation between the IC_50 _values for these peptides in cancer cells and normal cells was also assessed. The expression levels of TfR for the 12 cancer cell lines and two normal cell lines were assessed by flow cytometry using a PE-conjugated anti-TfR monoclonal antibody. As shown in Table 1, the MFI value for TfR monoclonal antibody in cancer cells ranged from 46.2 to 101.4, whereas the MFI for the normal cells ranged from 2.8 to 35.5. Thus, it was confirmed that these cancer cells express more TfR than normal cell lines, as reported previously (4-12). As shown in Figure 2, MFI value was not correlated with IC_50 _of TfR-lytic hybrid peptide (*r *= -0.67; Figure 2A) or lytic peptide alone (*r *= -0.47; Figure 2B). On the other hand, MFI value correlated well with the ratio of IC_50 _values between lytic peptide alone and TfR-lytic peptide in each cell line (*r *= 0.91; Figure 2C). These results suggest that the increase in cytotoxicity that occurs when the TfR moiety is combined to the lytic peptide is dependent on cell-surface TfR expression levels.

**Figure 3:**
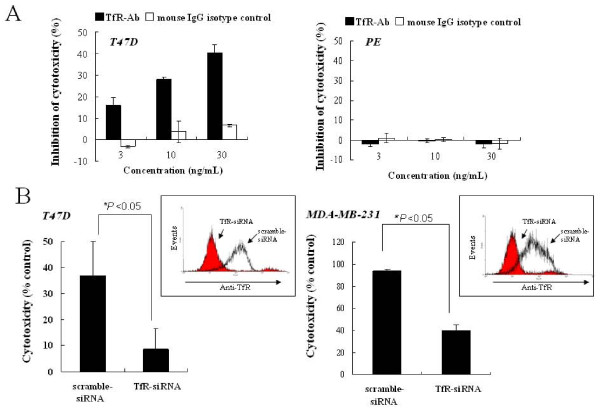
**Inhibition of cytotoxic activity of TfR-lytic hybrid peptide by anti-TfR antibody and knockdown using siRNA**. (A) T47D and PE cells were incubated with increasing concentrations of anti-TfR monoclonal antibody (TfR-Ab) or non-specific mouse IgG1 (isotype control) 3 h prior to TfR-lytic peptide treatment at 5 μM and 55 μM, respectively. (B) T47D or MDA-MB-231 cells were transfected with TfR-siRNA or scramble-siRNA, and 4 days after transfection, the levels of target protein in the cells were confirmed by flow cytometry analysis (inset graph). T47D and MDA-MB-231 cells were treated with TfR-lytic at 5 μM and 7.5 μM respectively. Inhibition rate was assessed using WST-8 reagent. Data are represented by means ± SD (bars) from triplicate determinations. Significance levels compared to the respective scramble-siRNA transfection: *, *P *< 0.05.

To further confirm the specificity of the TfR-lytic hybrid peptide to TfR, we performed a competition assay using anti-TfR monoclonal antibody and siRNAs specific to TfR. As shown in Additional file 1A, anti-TfR antibody used in this study was not toxic to normal and cancer cell lines (Additional file 1A). Either anti-TfR monoclonal antibody or mouse IgG isotype control was added to the T47D culture 3 h prior to the exposure of the TfR-lytic hybrid peptide to assess the effect on cytotoxic activity. As shown in Figure 3A, the inhibition of TfR-lytic-peptide cytotoxic activity in T47D cells by TfR monoclonal antibody was dose-dependent, whereas a significant difference in cytotoxic activity with regard to antibody concentration was not found with the mouse IgG isotype control. The inhibition of cytotoxic activity by anti-TfR or mouse isotype control antibody was not observed in normal cell lines, PE and HC (Figure 3A and Additional file 1B).

We also assessed whether cancer cells became less sensitive to TfR-lytic hybrid peptide on reduction of TfR expression by TfR-siRNA. The levels of target protein in the cells were confirmed by flow cytometry analysis (Figure 3B, inset graph). After transfection of TfR-siRNA into T47D or MDA-MB-231 cells, these cells became less sensitive to the cytotoxic effect of TfR-lytic hybrid peptide (5 μM for T47D cells and 10 μM for MDA-MB-231 cells) compared with that of cells transfected with the scramble-siRNA as a control (Figure 3B), and it was also confirmed that siRNA used in this study was not toxic to normal and cancer cell lines (T47D, MDA-MB-231, HC, and PE) as shown in additional file 2. These results suggest that the binding of the TfR-lytic hybrid peptide to TfR expressed on the cell surface is a critical requirement for the induction of target-specific cell killing.

### TfR-lytic hybrid peptide disintegrates the cancer cell membrane to induce rapid killing of cancer cells

**Figure 4:**
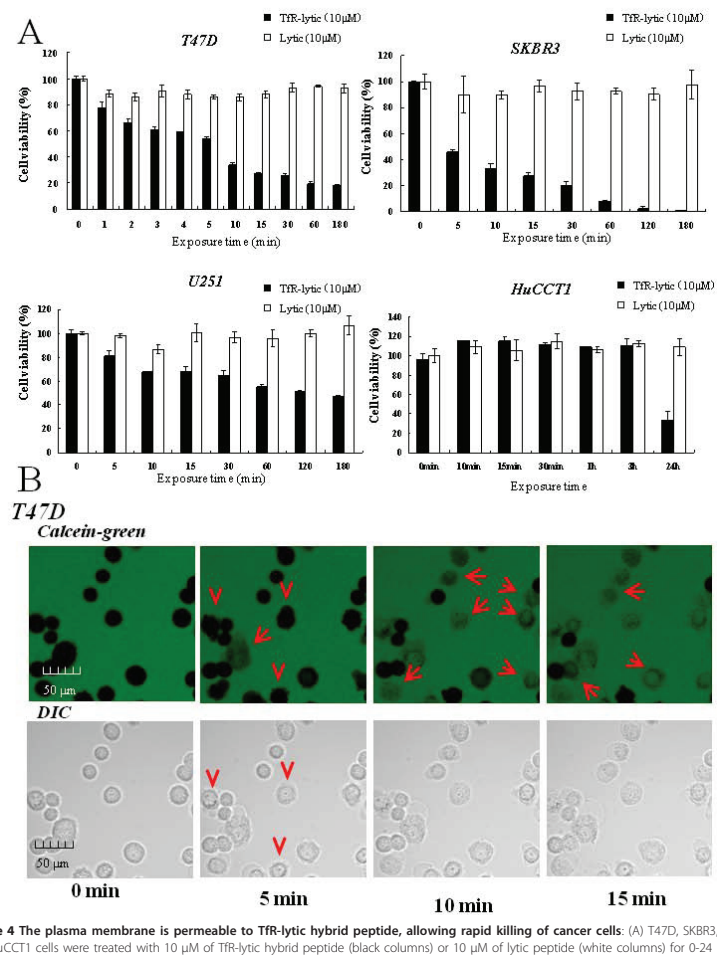
**The plasma membrane is permeable to TfR-lytic hybrid peptide, allowing rapid killing of cancer cells**: (A) T47D, SKBR3, U251, and HuCCT1 cells were treated with 10 μM of TfR-lytic hybrid peptide (black columns) or 10 μM of lytic peptide (white columns) for 0-24 hours, and the cells were analyzed for cell viability using WST-8 reagent. The results are represented as means ± SD (bars) from triplicate determinations. (B) Permeabilization of the cell membrane by TfR-lytic hybrid peptide in T47D breast cancer cells. Cells were observed in calcein solution 0, 5, 10, and 15 min after addition of TfR-lytic peptide (10 μM). Arrows indicate penetrated cells and arrowheads indicate a change in shape of the cell membrane by TfR-lytic peptide.

To explore the required duration of exposure to TfR-lytic hybrid peptide to kill cancer cells, T47D, SKBR3, BT20, U251, and HuCCT1 cells were treated with either TfR-lytic hybrid peptide or lytic peptide alone for 0-24 hours. As shown in Figure 4A and Additional file 3A, T47D, SKBR3 and BT20 cells, which were TfR overexpressed, maintained constant viability in the presence of lytic peptide (10 μM) for all exposure times. In contrast, the exposure of TfR-lytic hybrid peptide (10 μM) resulted in time-dependent loss of viability in these cells; a mere 10-min exposure of the TfR-lytic hybrid peptide to T47D, SKBR3, and BT20 cells was sufficient to kill more than 50% of cancer cells, and > 80% of cells lost viability after 60 min. On the other hand, U251 was induced more than 50% cell death at the concentration of TfR-lytic10 μM in approximately 3 hours, and it took more than 3 hours to induce enough cell death in HuCCT1. It is suggested that the speed of peptide accumulation to the cell membrane is slow in these TfR low-expressed cell lines (Figure 4A).

Further, using confocal fluorescence microscopy we assessed the interaction of TfR-lytic hybrid peptide with the cancer cell membrane. When T47D cells were cultured with the peptide (5 μM) for 0-15 min, a change of cell-membrane shape (Figure 4B, arrowheads) and an influx of calcein-labeled medium to the cancer-cell cytosol (Figure 4B, arrows) was observed within 5 min (Figure 4B). Ten minutes later, it was found that > 50% of cells had taken up calcein-labeled medium to the cytosol. Thus, TfR-lytic hybrid peptide disintegrates the cancer cell membrane that induces killing as a result of cell lysis. These results suggest that the TfR-lytic hybrid peptide kills cells very rapidly by a lytic mechanism upon binding to TfR.

### Characterization of the cancer cell death mechanism by TfR-lytic hybrid peptide

**Figure 5:**
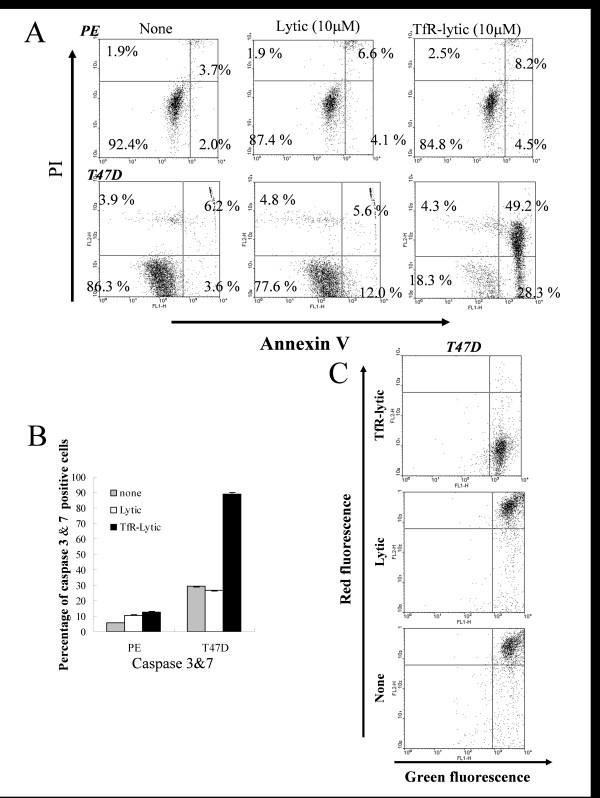
**Characterization of the cancer cell death mechanism by TfR-lytic hybrid peptide**: (A) Cancer T47D and normal PE cells were incubated with TfR-lytic peptide (10 μM) or lytic peptide (10 μM) for 3 h, then analyzed by dual-color flow cytometry for annexin V labeling and propidium iodide (PI) staining. (B) T47D and PE cells were incubated with TfR-lytic peptide (10 μM) or lytic peptide (10 μM) for 3 h, then analyzed by dual-color flow cytometry for caspase 3&7 and PI staining. Cell population values (%) are shown caspase 3&7-positive. The results are represented as means ± SD (bars) from triplicate determinations. (C) T47D cells labeled with the mitochondrial-transmembrane-potential-sensitive fluorescent dye JC-1 were treated with TfR-lytic peptide (upper panel) or lytic peptide (middle panel), or left untreated (lower panel), for 2 h, and analyzed for transmembrane potential by flow cytometry.

To determine whether the cytotoxic effect of TfR-lytic hybrid peptide was due to the cell death including an apoptotic cell death, cancer T47D and normal PE cells were treated with TfR-lytic peptide (10 μM) or lytic peptide alone (10 μM) for 3 h, and results were confirmed by annexin V-PI and caspase 3&7-PI staining using flow cytometry. As shown in Figure 5A, the externalization of phosphatidylserine, an early event in the apoptotic process, was analyzed with the annexin V-binding assay. Treatment of T47D cells with TfR-lytic peptide caused increase of annexin V positive cells (Figure 5A). Similar results were also obtained using other cell lines such as MDA-MB-231, BT20, SKBR3, which were TfR overexpressed cell lines as shown in additional file 3B, and TfR-lytic peptide induced Annexin positive cells to these cell lines from approximately 50 to 90 percent (Figure 5A and Additional file 3B), Whereas TfR-lytic peptide also Annexin positive cells to U251 and HuCCT1, which were TfR-low-expressed cell lines relatively, the percentage of induced Annexin positive cells by this hybrid peptide was lower than that of TfR overexpressed cell lines (Additional file 3A). On the other hand, the induction of apoptotic cell death was not observed in PE cells treated with TfR-lytic peptide.

In addition, caspase 3&7 was detected by DEVD-FMK covalent binding. As shown in Figure 5B, caspase 3&7 activation was detectable in T47D cells treated with TfR-lytic peptide; the population of caspase 3&7-positive cells was 89.1%. In contrast, the caspase 3&7-positive populations in PE cells treated with TfR-lytic peptide was 12.7%.

To further confirm the mechanism of apoptotic cell death by TfR-lytic peptide, mitochondrial dysfunction was assessed. T47D cells treated with TfR-lytic peptide or lytic peptide were evaluated for changes in the membrane potential of mitochondria by JC-1 staining. As shown in Figure 5C, exposure of T47D cells to TfR-lytic peptide resulted in a collapse of mitochondrial membrane potential, whereas exposure to lytic peptide alone had no influence on membrane potential. A tendency like T47D was also observed in MDA-MB-231 (Additional file 3C). These results suggest that TfR-lytic hybrid peptide induces cancer cell death by an apoptotic mechanism via caspase 3&7 activation.

### Antitumor activity of TfR-lytic peptide *in vivo*

**Figure 6:**
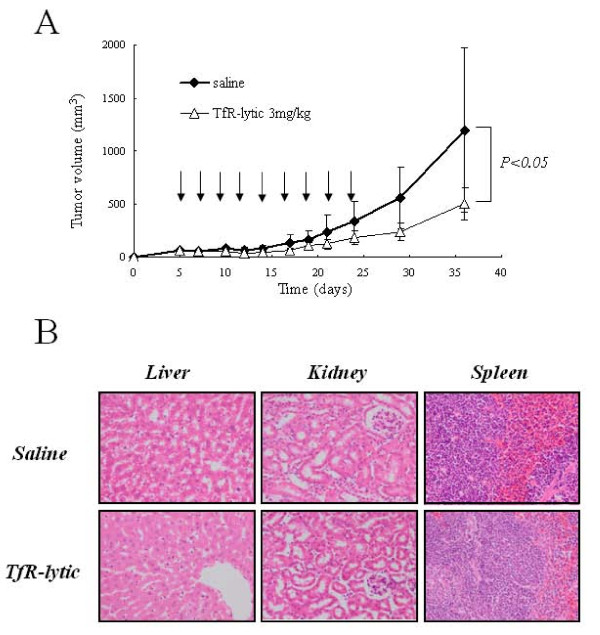
**Antitumour activity of TfR-lytic peptide in tumour xenograft model *in vivo***: (A) MDA-MB-231 breast cancer cells were implanted subcutaneously into athymic nude mice. Intravenous injection of either saline (control) or TfR-lytic peptide (3 mg/kg) was provided from day 5 as indicated by the arrows. Each group had six animals (n = 6), and experiments were repeated twice. Data are expressed as mean ± SD. (B) Histological examination after treatment with TfR-lytic peptide. Images (× 400 magnification) of liver, kidney and spleen from mice after treatment with saline (control) or TfR-lytic peptide (5 mg/kg) nine times were obtained by staining with hemaetoxylin and eosin (H&E).

To assess the antitumor effect of TfR-lytic peptide in a xenograft model of human cancer, MDA-MB-231 cells, which are TfR-overexpressing breast cancer cells were implanted subcutaneously into athymic mice. TfR-lytic peptide was injected intravenously at a dose of 3 mg/kg, three times a week for a total of nine doses. The tumor volume was inhibited significantly (*P *< 0.05) (Figure 6A). As shown in Figure 6A, The tumor volume of MDA-MB-231 on day 36 in the 3 mg/kg dosage group were reduced to 42% (500 mm^3^) of the control group with saline (1195 mm^3^). No abnormalities were observed in peripheral organs such as liver, kidney, and spleen in histological examination (Figure 6B). There were no differences in body weights and blood chemistries between the saline- and TfR-lytic peptide-treated groups (data not shown).

## Discussion

Therapeutic peptides are increasingly gaining popularity for medicinal use in a variety of applications [28], including tumor vaccines [29], antimicrobial therapy [30], and nucleic acid delivery [31]. TfR-binding peptide was identified as both diagnostic and potential therapeutic purposes by biopanning through sequential rounds of negative and positive selection [27]. Several peptides derived from phage-displayed peptide library screenings have been developed into drug candidates and tested in clinical trials, thus validating their peptide-targeting potential [32]. Following this research, the development of new peptide-based cancer therapeutics has been undertaken [33]. It is also known that peptide therapeutics are relatively easily generated using either recombinant or solid-phase chemical synthesis techniques and are generally less expensive than antibody-based therapeutics. In addition, because these peptides have lower molecular weights than proteins there is less steric hindrance and the advantage of improved target accessibility.

In this study we linked two functional domains to produce a novel bifunctional peptide that binds to TfR to cause lytic cell killing. It has been shown that the lytic sequence utilized in this study has higher selectivity than other lytic-type sequences thus far tested [25] in its discrimination between normal and cancer membranes and is suitable for chimerization with a targeting sequence. Accordingly, the cancer selectivity of TfR-lytic hybrid peptide designed in this study for TfR-expressing cancer cells was confirmed *in vitro *(Table 1 and Figure 2). Due to its targeting moiety, the hybrid peptide demonstrates selective killing of cancer cells as swiftly as 10 min after treatment (Figure 4A and 4B).

The TfR on normal cells is ubiquitously expressed at low levels and is expressed at greater levels on cells with a high proliferation rate, such as cells of the basal epidermis and intestinal epithelium [3-5]. Activated peripheral blood mononuclear cells express high levels of TfR [34-36]. In malignant tissues, TfR is expressed more abundantly than in normal counterpart tissue [37]. Therefore, TfR could be a relevant target for molecular targeted therapies against tumors.

The growth-inhibitory properties of anti-TfR antibodies have been pursued since the 1980s [38,39]. Various antibodies targeting TfR have shown different modes of action in different models, including blocking of transferrin binding to the receptor [40], blocking the internalization of the TfR-transferrin complex [41], downregulating cell-surface TfR [42], or causing intracellular degradation of TfR [43]. However, as Ng *et al. *indicated, all anti-TfR antibodies inhibit cell growth through iron deprivation [43]. Moura *et al. *[44] and Ng *et al. *[45] also showed that treatment of a neutralizing monoclonal antibody (mAb A24) and anti-TfR-avidin fusion protein (anti-rat TfR IgG3-Av) for 48 h and 96 h respectively, effectively inhibited proliferation of cancer cells. However, in this study, our data demonstrate that TfR-lytic peptide induces cancer cell-death as quickly as 10-15 min after treatment (Figure 4A and 4B). We hypothesize that the cytotoxic mechanism of TfR-lytic peptide is initiated by binding of the TfR-binding moiety of the hybrid peptide to TfR molecules on the cell surface, after which the lytic moiety of the hybrid peptide preferentially disintegrates the cancer cell membrane, induces mitochondrial damage, and triggers apoptotic cell death. Currently it is not clear how these lytic-type peptides induce apoptotic cell death on the cell surface. In this study, we demonstrated that TfR-lytic hybrid peptide induced annexin V-PI- and caspase 3&7-PI-positive cells, resulting in the collapse of mitochondrial membrane potential in cancer cells. Active caspases 3&7 are effector caspases activated by stimulation from mitochondria, cell-surface receptors, and endoplasmic reticulum, and by direct stimulation from stress-inducing molecules. In addition, we performed confocal fluorescence microscopy analysis and assessed the mitochondrial membrane potential by JC-1 staining to show that TfR-lytic peptide causes stimulation of the cell surface, suggesting that these cascades are all activated swiftly and simultaneously. Since TfR-lytic peptide quickly disintegrates the cell membrane and accumulates inside the cell, it is assumed that caspase cascades occur simultaneously when the hybrid peptide is administered. We measured the cytotoxic activity of TfR-lytic hybrid peptide to various cancer cell lines in which the expression levels of TfR are from high to low levels. The fold cytotoxic activation of COLO587 and HuCCT1 by TfR-lytic peptide was not so high, because the expression levels of TfR in these cells were low. The enhancement of cytotoxic activity by TfR-lytic peptide depends on the expression levels of TfR in cell lines, suggesting that TfR-lytic peptide is effectively targeted to cancer cell lines in which TfR is expressed dominantly. In addition, as we showed in Figure 4and additional file 3A, the speed of cancer cell killing by TfR-lytic peptide depends on the expression levels of TfR. We also previously showed that the designed lytic peptide was suitable for the conjugation to exert the enhancement of cytotoxic activity both *in vitro *and *in vivo *[26].

Although it is suggested that peptides are relatively easily inactivated by serum components in the human body, it has been shown that diastereomeric peptides are relatively free from inactivation in serum [46], and that a lytic diastereomeric peptide administered intravenously reduces tumor growth in an animal model of human prostate cancer without rapid degradation of the peptide in blood at a dose of 9 mg/kg [47]. Also, in our previous study, it was found that EGFR-lytic hybrid peptide targeting epidermal growth factor receptor (EGFR) administrated intravenously reduced the growth of EGFR-expressing tumor with a dose as low as 2 mg/kg [26]. IC_50 _of TfR-lytic is 5-10 μM *in vitro*, which is approximately 18.5 to 37 mg/L. Given that a total blood volume of the nude mice of about 20 g (body weight) is 1.5 ml, we expected that TfR-lytic may exert enough antitumor effects at a dose of approximately 2.8 to 5.6 mg/kg. In this *in vivo *study, TfR-lytic-treated group showed 42% of tumor growth-inhibitory effect at a dose of 3 mg/kg. We demonstrated that this dose coincide with that of expected from *in vitro *data.

So far, several drug candidates, including TfR antibody 42/6 and TfR-diphtheria immunotoxin, have shown limited antitumor activity without severe side effects in clinical trials [48-50]. We expect that TfR-lytic hybrid peptide may offer new options for TfR-targeted cancer therapies. Standard therapy for malignant gliomas usually includes surgical debulking or biopsy, external beam radiation therapy, and systemic chemotherapy. These treatments are incomplete because some tumor cells are allowed to survive, leading to tumor progression or recurrence. TfR-lytic peptide, like all targeted cytotoxins, offers the possibility of targeting these refractory tumor cells because malignant glioma cells express TfR [50]. Our current *in vitro *results have shown a clear dependence of the drug on the TfR moiety, suggesting high selectivity for tumor cells and less cytotoxity toward normal cells. This selective targeting ability should provide a large therapeutic window of opportunity for targeting cancer cells over normal cells. Further studies to confirm its efficacy, safety, and immunogenicity will broaden the indications of TfR-lytic hybrid peptide for the future.

## Conclusions

It was found that TfR-lytic peptide binds specifically to TfR and selectively kills cancer cells and suggested that TfR-lytic peptide penetrates the cancer cell membrane, and induces rapid killing and apoptotic cell death. Furthermore, the intravenously administration of TfR-lytic peptide in the athymic mice model significantly inhibited tumor progression. TfR-lytic peptide might provide a potent and selective anticancer therapy for patients.

## Competing interests

Masayuki Kohno is an employee, and Koji Kawakami is a founder and stock holder of Upstream Infinity, Inc. The other authors disclose no potential conflicts of interest.

## Authors' contributions

MK carried out all of the experiments and wrote this manuscript. HT and MK designed the hybrid peptide, and contributed to data analysis and the writing of the manuscript. KK contributed to the conception and design of the study. All authors read and approved the final manuscript.

## Pre-publication history

The pre-publication history for this paper can be accessed here:

http://www.biomedcentral.com/1471-2407/11/359/prepub

## Supplementary Material

Additional file 1 **Effect of anti-TfR antibody on the cell viability of T47D, HC, and PE cells**. (A) T47D, HC and PE cells were incubated with increasing concentrations of anti-TfR monoclonal antibody (TfR-Ab) for 3 h, and the cytotoxicity was assessed using WST-8 reagent. Data are represented by means ± SD (bars) from triplicate determinations. There was no statistical difference in the viability between none treatment and treatment with TfR-Ab 30 ng/mL. (B) HC cells were incubated with increasing concentrations of anti-TfR monoclonal antibody (TfR-Ab) or non-specific mouse IgG1 (isotype control) 3 h prior to TfR-lytic peptide treatment at 55 μM. Inhibition rate of the cytotoxic activity was assessed using WST-8 reagent. Data are represented by means ± SD (bars) from triplicate determinations. Click here for file

Additional file 2 **Effect of knockdown of TfR by siRNA on the cell viability of T47D, MDA-MB-231, HC and PEcells**. (A) T47D, MDA-MB-231, HC, and PE cells were transfected with TfR-siRNA scramble-siRNA or none, and the cytotoxicity was assessed using WST-8 reagent. Data are represented by means ± SD (bars) from triplicate determinations. There was no statistical difference in the viability between none treatment and treatetment with scramble- and TfR-siRNA. (B) HC and PE cells were transfected with TfR-siRNA or scramble-siRNA, and 4 days after transfection, cell viability was assessed using WST-8 reagent. Data are represented by means ± SD (bars) from triplicate determinations. Click here for file

Additional file 3 **Characterization of cancer cell death induced by TfR-lytic hybrid peptide**. (A) BT20 cells were treated with the 10 μM of TfR-lytic hybrid peptide (black columns) or 10 μM of lytic peptide (white columns) for 0-180 min, and the cells were analyzed for cell viability using WST-8 reagent. The results are represented as means ± SD (bars) from triplicate determinations. (B) MDA-MB-231, SKBR3, BT20, U251, and HuCCT1 cells were incubated with TfR-lytic peptide (10 μM) and lytic peptide (10 μM) for 3 h, and then analyzed by dual-color flow cytometry for annexin V labeling and propidium iodide (PI) staining.(C) MDA-MB-231 cells labeled with the mitochondrial-transmembrane-potential-sensitive fluorescent dye JC-1 were treated with TfR-lytic peptide (right panel) or lytic peptide (middle panel), or left untreated (left panel), for 2 h, and analyzed for transmembrane potential by flow cytometry. Click here for file
