# Supplementary figures and images for: A novel transferrin receptor-targeted hybrid peptide disintegrates cancer cell membrane to induce rapid killing of cancer cells
Source: BMC Cancer. 2011 Aug 18;11:359. doi: 10.1186/1471-2407-11-359 (PMC3167775; doi:10.1186/1471-2407-11-359)

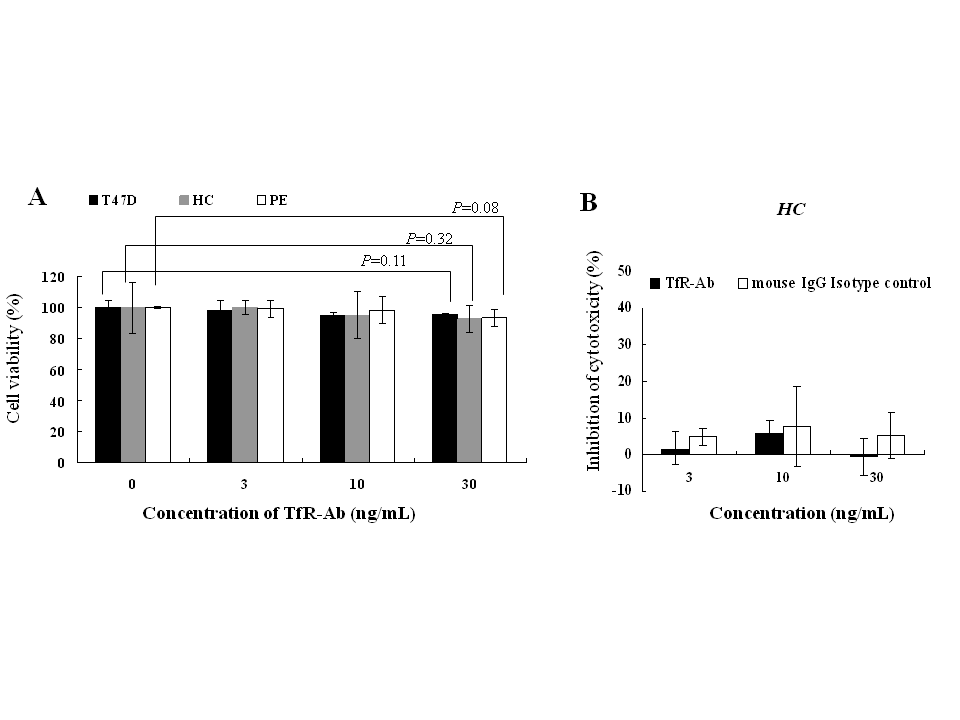

Supplement: Additional file 1 — Effect of anti-TfR antibody on the cell viability of T47D, HC, and PE cells. (A) T47D, HC and PE cells were incubated with increasing concentrations of anti-TfR monoclonal antibody (TfR-Ab) for 3 h, and the cytotoxicity was assessed using WST-8 reagent. Data are represented by means ± SD (bars) from triplicate determinations. There was no statistical difference in the viability between none treatment and treatment with TfR-Ab 30 ng/mL. (B) HC cells were incubated with increasing concentrations of anti-TfR monoclonal antibody (TfR-Ab) or non-specific mouse IgG1 (isotype control) 3 h prior to TfR-lytic peptide treatment at 55 μM. Inhibition rate of the cytotoxic activity was assessed using WST-8 reagent. Data are represented by means ± SD (bars) from triplicate determinations. [file 1471-2407-11-359-S1.TIFF]

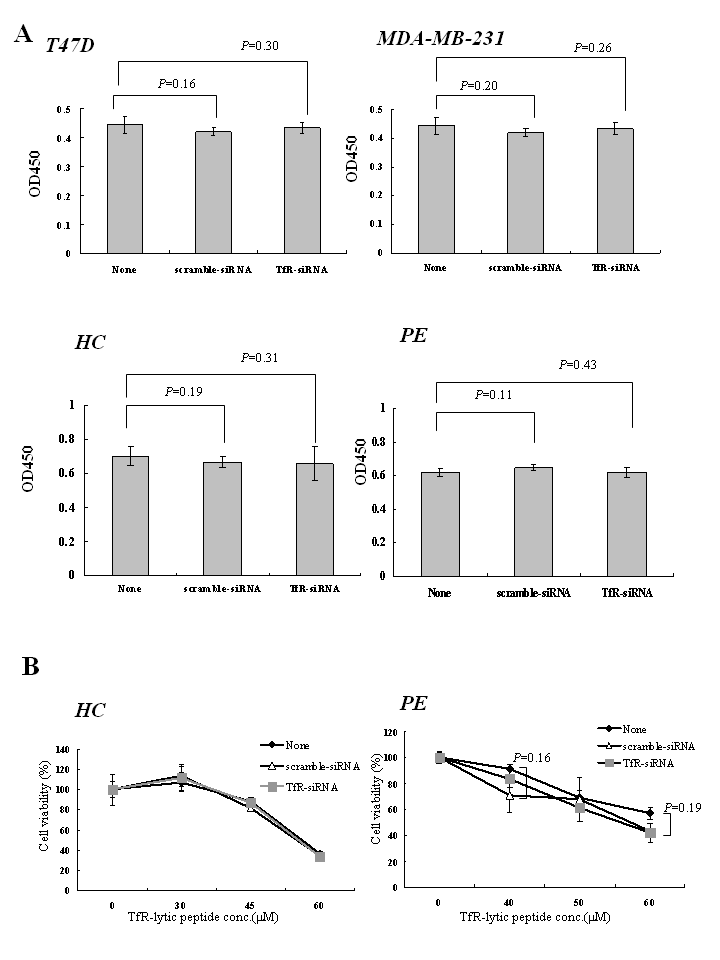

Supplement: Additional file 2 — Effect of knockdown of TfR by siRNA on the cell viability of T47D, MDA-MB-231, HC and PEcells. (A) T47D, MDA-MB-231, HC, and PE cells were transfected with TfR-siRNA scramble-siRNA or none, and the cytotoxicity was assessed using WST-8 reagent. Data are represented by means ± SD (bars) from triplicate determinations. There was no statistical difference in the viability between none treatment and treatetment with scramble- and TfR-siRNA. (B) HC and PE cells were transfected with TfR-siRNA or scramble-siRNA, and 4 days after transfection, cell viability was assessed using WST-8 reagent. Data are represented by means ± SD (bars) from triplicate determinations. [file 1471-2407-11-359-S2.TIFF]

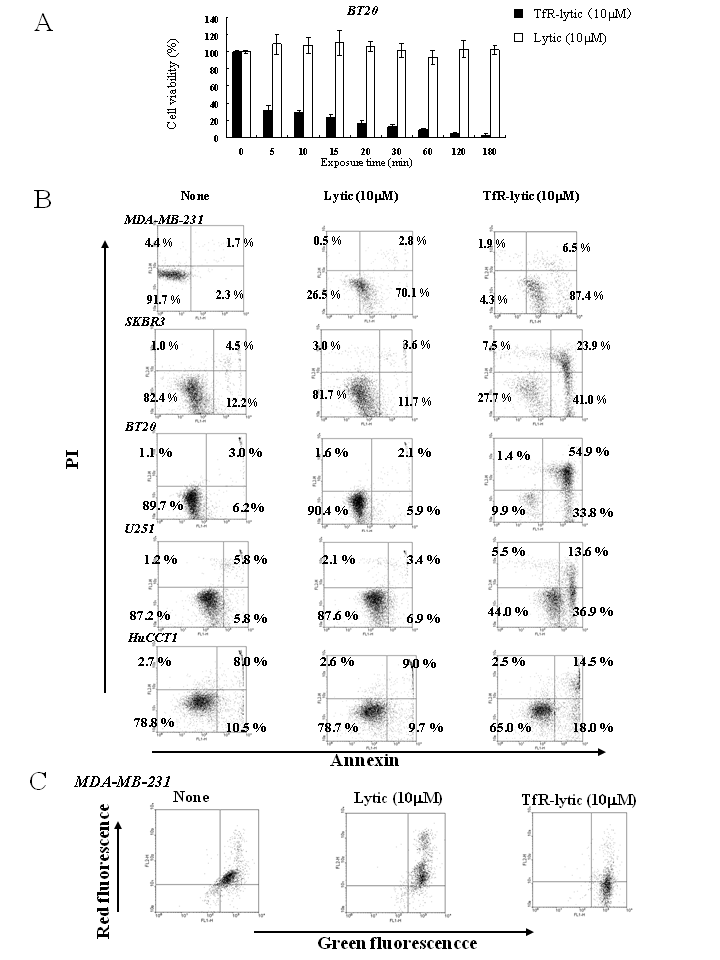

Supplement: Additional file 3 — Characterization of cancer cell death induced by TfR-lytic hybrid peptide. (A) BT20 cells were treated with the 10 μM of TfR-lytic hybrid peptide (black columns) or 10 μM of lytic peptide (white columns) for 0-180 min, and the cells were analyzed for cell viability using WST-8 reagent. The results are represented as means ± SD (bars) from triplicate determinations. (B) MDA-MB-231, SKBR3, BT20, U251, and HuCCT1 cells were incubated with TfR-lytic peptide (10 μM) and lytic peptide (10 μM) for 3 h, and then analyzed by dual-color flow cytometry for annexin V labeling and propidium iodide (PI) staining.(C) MDA-MB-231 cells labeled with the mitochondrial-transmembrane-potential-sensitive fluorescent dye JC-1 were treated with TfR-lytic peptide (right panel) or lytic peptide (middle panel), or left untreated (left panel), for 2 h, and analyzed for transmembrane potential by flow cytometry. [file 1471-2407-11-359-S3.TIFF]
